# The effect of puppyhood and adolescent diet on the incidence of chronic enteropathy in dogs later in life

**DOI:** 10.1038/s41598-023-27866-z

**Published:** 2023-02-09

**Authors:** Kristiina A. Vuori, Manal Hemida, Robin Moore, Siru Salin, Sarah Rosendahl, Johanna Anturaniemi, Anna Hielm-Björkman

**Affiliations:** 1 Faculty of Veterinary Medicine, Department of Equine and Small Animal Medicine, University of Helsinki, Helsinki, Finland; 2 Department of Nutrition and Clinical Nutrition, Faculty of Veterinary Medicine, Beni-Suef University, Beni-Suef, Egypt; 3 Department of Agricultural Sciences, Faculty of Agriculture Forestry, University of Helsinki, Helsinki, Finland

**Keywords:** Colonic diseases, Diarrhoea, Inflammatory bowel disease, Irritable bowel syndrome, Irritable bowel syndrome, Gastroenteritis, Inflammatory bowel disease, Chronic inflammation, Inflammatory diseases, Risk factors, Digestive signs and symptoms, Gastroenterology, Gastrointestinal system

## Abstract

Diet has a key role in the homeostasis of the gut microenvironment, influencing the microbiome, the gut barrier, host immunity and gut physiology. Yet, there is little information on the role of early diet in the onset of inflammatory gastrointestinal disorders later in life, especially in dogs. Therefore, the aim of the present cross-sectional, epidemiological study with longitudinal data, was to explore associations of companion dogs’ early life diet style and food items with owner-reported chronic enteropathy (CE) incidence in later life. Food frequency questionnaire data from Finnish companion dogs was analyzed using principal component analysis and logistic regression. We found that feeding a non-processed meat-based diet and giving the dog human meal leftovers and table scraps during puppyhood (2–6 months) and adolescence (6–18 months) were protective against CE later in life. Especially raw bones and cartilage as well as leftovers and table scraps during puppyhood and adolescence, and berries during puppyhood were associated with less CE. In contrast, feeding an ultra-processed carbohydrate-based diet, namely dry dog food or “kibble” during puppyhood and adolescence, and rawhides during puppyhood were significant risk factors for CE later in life.

## Introduction

The health of humans, animals and the environment is inextricably linked as recognized by the One Health Initiative^1^. Companion dogs and humans in middle- and high-income countries suffer from the same non-communicable diseases including chronic inflammatory gastrointestinal disorders^2–4^. Canine chronic enteropathies (CE)^5^ and human inflammatory bowel diseases (IBD) share many similarities^6,7^. Canine CE gastrointestinal symptoms include persistent and/or recurrent vomiting, diarrhea, intestinal sounds and gas, decreased appetite, abdominal pain, nausea and/or weight loss which last longer than three weeks^5–7^. The symptoms have severe and stressful impacts on the dog’s life^8^ and increase the caregiver burden of the owner^9^.

The multifactorial etiopathogenesis of both canine CE and human IBD remains elusive. Current knowledge suggests the importance of interactions between the gut microenvironment (microbiota and diet composition) and the host immune system^10,11^. Common causes in both species include genetic predisposition^12–15^, lower microbial diversity (species richness and evenness) of the GI microbiota^11,16,17^ as well as a diet containing high amounts of processed foods^12,18^. Chronic enteropathy and IBD are also associated with low levels of certain micronutrients such as vitamin D^19,20^.

The diet is the source of nutrients and impacts microbial composition in health and disease^17,21–23^. Research regarding the role of the early diet on CE incidence in dogs is scarce. Hemida et al. recently reported a protective influence on the prevalence of CE when the puppies were consuming a non-processed meat-based diet and a predisposing effect when they were consuming an ultra-processed carbohydrate-based diet during the early and late postnatal periods^12^. In humans, the Western diet which contains different ultra-processed foods and high amounts of sugar has been connected to IBD risk^24^. Thus a greater understanding of dietary choices and dietary components that are a risk or can have a protective effect can help in preventing the disease. As early dietary exposures are modifiable, the dog owners would then have a chance to act proactively and have an impact on their dog's health.

The aim of the present cross-sectional, longitudinal epidemiological study was to explore associations of companion dogs’ early life diet style and their consumed food items, with owner-reported CE incidence in later life. We hypothesized that both diet style and eating certain food items during puppyhood and adolescence would influence future CE incidence. A wide range of food items were covered in the study. Analyses of diet style, inferred from several food variables using a multivariate method, as well as feeding frequencies of single food items and other edible items were surveyed.

## Materials and methods

### The questionnaire and study sample

The study data was extracted from the online DogRisk food frequency questionnaire (FFQ) data in 2019 and included a total of 16,607 answers. The FFQ is an epidemiological, owner-reported cross-sectional questionnaire, established in 2009 at the University of Helsinki. It has an ethical approval (29.4.2016) from the University of Helsinki Viikki campus ethical board. A waiver for informed consent was obtained from the Research Ethics Committee on Animal Research at the Helsinki University, Finland. Details of the FFQ have been previously published elsewhere^12,25,26^. In brief, the validated FFQ includes questions about the frequency (never, a couple of times per year, a couple of times per month, a couple of times per week, always or almost always) of feeding different food items to the dog or of the dog eating items outdoors, at three different life stages (puppyhood (PU), adolescence (YO), adult), together with a range of questions about the dog’s phenotype, environment, health status, lifestyle and maternal data. Accuracy of owner-reported CE has not been evaluated. However, three diagnoses out of the 117 diagnoses in the DogRisk questionnaire were evaluated in the validation study; canine atopic dermatitis, hip dysplasia and hypothyroidism were compared to veterinary diagnoses, official radiographic scores, and concurrent thyroid medication, respectively, showing high accuracy (91–95%) of the owners’ disease reporting^26^. Based on this and literature, we use “CE” as an acronym for the owner-reported chronic enteropathy throughout the text: “CE can be used for animals in which intestinal inflammation is suspected but has not been documented (i.e. no biopsies have been taken)”^7^. CE is nowadays a common name for a more than three week long GI disease that can either be treated with diet, antibiotics or cortisone, showing its etiological diversity^5,7^. All methods were performed in accordance with the relevant guidelines and regulations.

**Figure 1:**
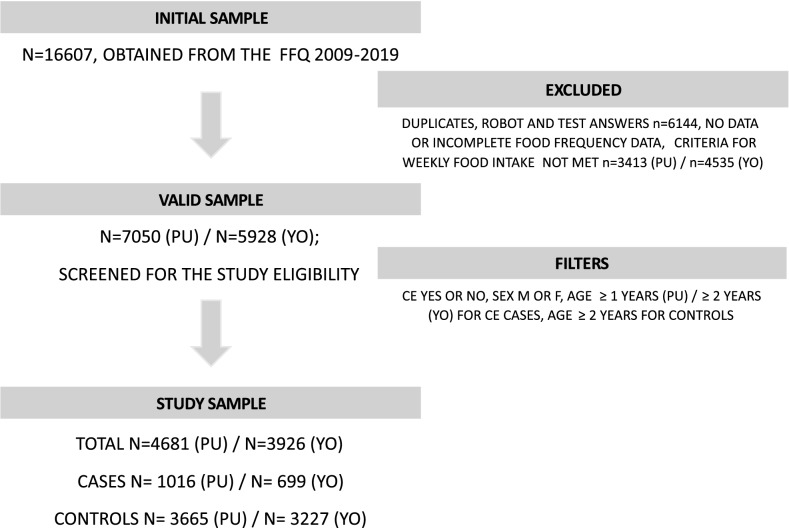
Flow chart of the study sample. PU = puppyhood, YO = adolescent, CE = owner-reported chronic enteropathy, M = male, F = female, FFQ = food frequency questionnaire.

Test, robot and duplicate answers (n = 6144) were first removed (Fig. 1). The feeding part of the FFQ included questions about 47 food items of which 21 were possible to answer choosing two different items from dropdown menus. Seven more questions were related to items that the dog eats outside, whereof one was possible to answer choosing two different items. The answers were grouped into 53 food items with similar nutrition content and/or processing procedure (Supplementary information 1) and the maximum feeding frequencies were used as the final test variables. After excluding variables with > 50% missing data, we used 46 food item variables (Supplementary information 1). A sum variable of energy containing feeds (Supplementary information 1) was calculated for each dog at the different life stage categories. If the dog’s data was missing for the studied age period or detailed food frequency questions or did not include energy-containing feeds at least 5 times per week, the dog’s data on that life stage category was excluded from the analyses (n = 3413 (PU; 2–6 months)/n = 4535 (YO; 6–18 months)). Thus, the valid samples for the age categories PU and YO were N = 7050 and N = 5928, respectively (Fig. 1). The datasets used and/or analysed during the current study are available from the corresponding author on reasonable request.

We performed principal component analyses (PCA) for PU and YO valid population datasets using the package psych^27^ in R (r.org) with polychoric correlation matrix and varimax rotation. Threshold values for variable loadings ( >|0.4|) and communalities (> 0.3) were used. For PCA, missing values were imputed with null (corresponding to the answer “never”). Selected component scores for feeding styles were used in further analyses.

There were seven questions related to CE in the FFQ: “Has your dog suffered from inflammatory bowel disease (IBD), chronic gastrointestinal symptoms and/or food ‘allergies’ resulting in chronic gastrointestinal symptoms? (Yes/No)”/“The frequency of the symptoms? (Rarely/Often)”/“The starting age of the symptoms? (Dropdown for Years and Months separately)”/“The dog is currently affected by the disease. (Checkbox)”/“The symptoms ended after a diet change. (Checkbox)”/ “A diet change did not alleviate the symptoms. (Checkbox)”. If the owner answered “Yes” to the first question and/or in ≥ two other CE related questions, the dog was considered as a case. If the owner answered “No” to the first question and/or gave < two answers to the other CE related questions, the dog was considered as a control. We use “CE” as an acronym for the owner-reported chronic enteropathy throughout the text (“CE can be used for animals in which intestinal inflammation is suspected but has not been documented (i.e. no biopsies have been taken)”^7^). To avoid reverse causality, in the PU data cases ≥ one year and controls ≥ two years old were selected. In the YO data, cases and controls ≥ two years old were selected. After selection, the study samples included n = 1016 (PU)/n = 699 (YO) cases and n = 4709 (PU)/n = 4171 (YO) controls.

We studied the effect of the general feeding style as PCA component scores (RC1 non-processed meat-based diet in inverse association with ultra-processed carbohydrate-based food, RC2 home cooked diet, RC3 human food leftovers and table scraps) on later CE incidence as binary dependent variables using multivariate logistic regression. We also transformed the PCA scores of significant (*p* < 0.05) feeding styles RC1 (min–max − 1.305 to 3.8205) and RC3 (min–max − 2.624 to 3.902) to correspond the original feeding frequency units (0–4) and did additional analysis of the effects of frequencies. A Mann–Whitney U-test was done to preliminary screen food items that were significantly (*p* < 0.05) different between case and control groups (Supplementary information 2). Backward stepwise regression with entry probability ≤ 0.05 and removal probability ≥ 0.10 was used for further investigation of the effects of individual food items within a feeding style on later CE incidence. Only items that were significantly different in the preliminary Mann–Whitney U-tests were included. Multicollinearity was checked by variance inflation factors (VIF, criteria < 4.0) and tolerance (criteria > 0.2). The model fit quality was investigated by the Hosmer and Lemeshow test (*p* value > 0.05). The variables with *p* < 0.05 were considered statistically significant. Models were adjusted for gender as it had a significant effect on CE incidence. Age was not used in the models as the effect was not significant. We also tested the significances of feeding styles by adding earlier reported “IBD prone breeds”, gathered from the literature and presented in Hemida et al. (2021)^12^ to the models for further adjustment. However, we opt to present the model results as simple as possible for clarity and thus without additional adjustments and only mention when the effect retained significance when the model was further adjusted. IBM SPSS Statistics software (version 27) was used for logistic regression and Mann–Whitney U-tests.

## Results

### Chronic enteropathy prevalence and sample characteristics in the Food Frequency Questionnaire responses

Prevalence of owner-reported CE symptoms was 21.7% in the puppies (2–6 months) and 17.8% in the adolescent dogs (6–18 months). Owners reported onset of CE symptoms at 1.4 years mean age (n = 913, min–max 0.2–12 years, standard deviation 1.6) in the PU data and at 1.5 years mean age (n = 599, min–max 0.2–12 years, standard deviation 1.6) in the YO data. The average age of the controls was 5.4/5.5 years in PU and YO data, and the average age of the cases was 4.5/5.4 years in PU and YO data, respectively (Table 1). The gender ratios within the controls and cases were 56.9% females/43.1% males and 40.5% females/59.5% males in the PU data, and 57.2% females/42.8% males and 37.8% females/62.2% males in the YO data, respectively (Table 1). Breed information^12^ was available only for part of the dogs (85.4–86.0%). For that part, the ratio of non-prone breeds vs prone breeds was 49.1%/50.9% in the PU data and 50.2% /49.8% in the YO data (Table 1). Also, 94% of the YO-dogs were the same dogs that already were analysed in the PU data.

**Table 1:** Characteristics of the study sample.

	Puppyhood		Adolescence	
	Controls	Cases	Controls	Cases
	n = 3665	n = 1016	n = 3227	n = 699
Age (years)	5.4	4.5	5.5	5.4
Gender				
Female	56.9%	40.5%	57.2%	37.8%
Male	43.1%	59.5%	42.8%	62.2%
Breed	n = 3138	n = 860	n = 2778	n = 598
CE prone	51.7%	48.3%	49.8%	52.2%
Non-prone	52.2%	47.8%	50.2%	47.8%

### Diet style during puppyhood and adolescence influences the incidence of chronic enteropathy later in life

The food items’ loadings onto PCA components were identical in the PU and YO datasets (Table 2). We used selected component scores RC1 (non-processed foods in inverse association with ultra-processed food; abbreviated as NPMD + UPCD-), RC2 (home cooked food; abbreviated as Cooked) and RC3 (human food leftovers and table scraps; abbreviated as Leftovers) for investigating the effect of general diet style on CE. The component “RC1” included all the questionnaire’s food items belonging to a non-processed meat-based diet, NPMD (raw red meat, organ meats, fish, eggs, tripe, bones and cartilage, vegetables, berries and fruits) and fat supplements (fish and vegetable oils, animal fat) in an inverse association with dry dog food which is an ultra-processed and carbohydrate-based diet, UPCD. The inverse association of non-processed foods and dry dog food in the RC1 component means that owners gave both foods in a reciprocal manner so that when the amount of non-processed meat-based food items given to the dog was high, the amount of dry dog food given was low, and vice versa. All (PU) and the majority of the (YO) non-processed food items within the RC1 component were also significantly different between the CE cases and controls in the preliminary Mann–Whitney U-tests (Table 2 and Supplementary information 2).

**Table 2:** Food items' loadings to PCA components showing puppyhood (PU) and adolescent (YO) diet style.

Puppyhood	RC1 NPMD + UPCD- 17%	RC2 Cooked 12%	RC3 Leftovers 12%	RC4 Outside 11%	Adolescence	RC1 NPMD + UPCD- 17%	RC2 Cooked 13%	RC3 Leftovers 12%	RC4 Outside 10%
**Raw berries**	**0.54**	0.11	0.22	0.26	**Raw berries**	**0.58**	0.08	0.2	0.23
**Raw vegetables**	**0.64**	0.09	0.2	0.18	**Raw vegetables**	**0.68**	0.07	0.15	0.15
**Cooked vegetables**	0.27	**0.63**	0.39	0.04	**Cooked vegetables**	0.25	**0.67**	0.36	0.04
**Cooked potato**	0.14	0.22	**0.68**	0.11	**Cooked potato**	0.07	0.18	**0.71**	0.13
**Fruits**	**0.48**	0.18	0.26	0.16	*Fruits*	**0.5**	0.18	0.25	0.15
*Cooked tripe*	0.1	**0.78**	− 0.01	0.08	*Cooked tripe*	0.05	**0.82**	− 0.03	0.07
**Raw tripe**	**0.78**	0.08	− 0.01	0.05	**Raw tripe**	**0.77**	0.07	− 0.06	0.05
**Cooked organ meats**	0.25	**0.83**	0.07	0.07	**Cooked organ meats**	0.23	**0.84**	0.06	0.08
**Raw organ meats**	**0.88**	− 0.05	− 0.03	0.06	**Raw organ meats**	**0.87**	− 0.05	− 0.1	0.07
*Cooked bone and cartilage*	0.06	**0.78**	0.06	0.12	*Cooked bone and cartilage*	0.02	**0.78**	0.06	0.14
**Raw bone and cartilage**	**0.80**	0.05	0.04	0.13	**Raw bone and cartilage**	**0.81**	0.01	− 0.01	0.13
**Non-sour milk products**	0.09	0.1	**0.65**	0.15	**Non-sour milk products**	0.05	0.1	**0.64**	0.15
*Vegetable oils*	**0.48**	0.2	0.32	0.1	*Vegetable oils*	**0.53**	0.19	0.25	0.06
*Fish oils*	**0.56**	0.2	− 0.02	0.02	*Fish oils*	**0.54**	0.16	− 0.01	0.01
*Animal fats*	**0.46**	0.14	0.35	0.04	*Animal fats*	**0.46**	0.14	0.31	0.03
**Cooked egg**	0.21	**0.62**	0.34	0.08	**Cooked egg**	0.19	**0.65**	0.32	0.08
**Raw egg**	**0.7**	0.03	0.14	0.1	**Raw egg**	**0.72**	0.02	0.1	0.09
**Dry dog food**	− **0.72**	− 0.01	− 0.02	0.12	*Dry dog food*	− **0.73**	− 0.01	0.12	0.11
**Blood pancakes**	0.22	0.16	**0.56**	0.08	**Blood pancakes**	0.19	0.15	**0.57**	0.09
*Liver casserole*	− 0.02	0.16	**0.64**	0.01	**Liver casserole**	− 0.04	0.15	**0.64**	0.04
**Human meal leftovers**	0.05	0.02	**0.73**	0.18	**Human meal leftovers**	0	0.02	**0.72**	0.19
**Grain products**	0.23	**0.4**	**0.57**	0.14	*Grain products*	0.17	**0.44**	**0.53**	0.09
*Cooked rice*	0.14	**0.51**	**0.49**	0.1	**Cooked rice**	0.17	**0.5**	**0.41**	0.1
**Raw red meat**	**0.57**	0.1	0.03	0.11	*Raw red meat*	**0.6**	0.1	0.03	0.07
*Cooked red meat*	− 0.09	**0.71**	0.32	0	*Cooked red meat*	− 0.1	**0.7**	0.34	0
*Cooked poultry*	− 0.13	**0.58**	**0.46**	0	*Cooked poultry*	− 0.13	**0.52**	**0.46**	0.02
*Processed meat*	− 0.05	0.06	**0.52**	0.21	*Processed meat*	− 0.07	0.11	**0.52**	0.18
**Cooked fish**	0.11	**0.45**	**0.47**	0.05	**Cooked fish**	0.08	**0.42**	**0.47**	0.05
**Raw fish**	**0.84**	− 0.05	− 0.06	0.07	*Raw fish*	**0.79**	− 0.11	− 0.1	0.09
**Sticks outside**	− 0.01	0.06	0.12	**0.74**	**Sticks outside**	0.01	0.06	0.1	**0.71**
*Grass outside*	0.09	0.07	0.16	**0.76**	*Grass outside*	0.09	0.08	0.14	**0.73**
**Clay and stones outside**	0.03	0.06	0.02	**0.77**	*Clay and stones outside*	0.02	0.06	0.03	**0.77**
**Dirt outside**	0.06	0.08	0.04	**0.79**	*Dirt outside*	0.05	0.11	0.05	**0.78**
**Puddles outside**	0.16	0.01	0.18	**0.68**	**Puddles outside**	0.15	0.03	0.19	**0.67**
*Feces outside*	0.16	0.07	0.09	**0.58**	*Feces outside*	0.14	0.07	0.11	**0.59**
**Carcasses outside**	0.25	0.01	0.23	**0.53**	**Carcasses outside**	0.2	0.01	0.23	**0.58**

Variables that were significantly different (Mann Whitney U-test *p* < 0.05) between controls and CE cases in the preliminary screening are in bold. Loadings ≥ |0.4| are in bold. NPMD = non-processed meat-based diet, UPCD = ultra-processed carbohydrate-based diet, Cooked = home-cooked diet, Leftovers = human meal leftovers and table scraps, Outside = items eaten outside. Percent of variation explained is shown for each component.

The component “RC2” included cooked ingredients (cooked red meat, poultry, organ meats, fish, eggs, tripe, bones and cartilage, vegetables, rice and grain products) likely offered as a home-cooked meal for the dog. Half of the items in this component were not significant in the preliminary Mann–Whitney U-tests between CE cases and controls (Table 2 and Supplementary information 2).

The component “RC3” included human meal leftovers and table scraps (leftovers, cooked potato, non-sour milk products, cooked poultry and fish, processed meat (for example sausages), cooked rice and grain products, blood pancakes and liver casserole (the latter two are two convenience foods often given to dogs in Finland)). RC2 and RC3 share some items such as cooked fish, poultry, rice and grain products. Most of the items in this component were significantly different between the CE cases and the controls in the preliminary Mann–Whitney U-tests (Table 2 and Supplementary information 2).

**Figure 2:**
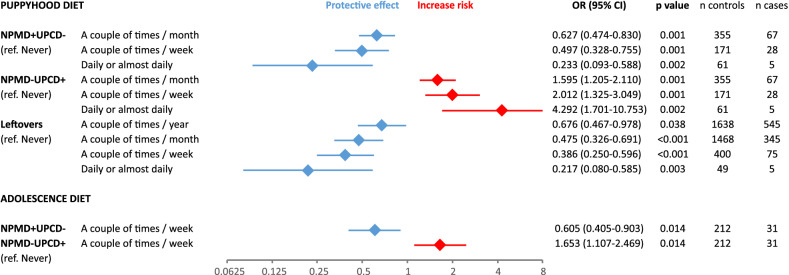
Odds ratios (OR) of the significant (*p* < 0.05) associations between the puppyhood and adolescence diet feeding frequencies and the incidence of chronic enteropathy (CE) in adult dogs based on multivariate logistic regression analyses (adjusted for gender). The inverse association of NPMD (non-processed meat-based diet) and UPCD (ultra-processed carbohydrate-based diet) means that owners gave both foods in a reciprocal manner so that when the amount of NPMD given for the dog was high, the amount of UPCD given was low and vice versa. Red color denotes increased risk, blue color denotes protective effect, CI = confidence interval, ref = reference.

Eating a non-processed meat-based diet (high RC1 score) during PU and YO had a significantly protective effect and eating an ultra-processed carbohydrate-based diet (low RC1 score or RC1 score inverted) had a significantly predisposing effect on the future CE incidence (logistic regression, *p* < 0.001 and *p* = 0.008, for PU and YO respectively) (Table 3). These effects were more pronounced in the PU data. Eating human meal leftovers and table scraps during PU and YO had a significantly protective effect on the future CE incidence (logistic regression, *p* < 0.001) whereas the home-cooked meal diet (RC2 score) did not have a significant effect (Table 3). The protective effects of a NPMD and leftovers as well as the predisposing effect of UPCD retained statistical significances also when the models were further adjusted with “IBD prone breed” (data not shown). The odds of CE decreased significantly (*p* < 0.05) when the feeding frequency of NPMD increased during PU i.e. when the NPMD was given a couple of times per month or more often (Fig. 2). By contrast, the odds of CE increased significantly (*p* < 0.05) when the feeding frequency of UPCD increased during PU i.e. when the UPCD was given a couple of times per month or more often (Fig. 2). Odds of CE also decreased significantly (*p* < 0.05) when the feeding frequency of leftovers increased during PU (Fig. 2). Feeding NPMD a couple of times in a week during YO had a significantly (< 0.05) decreasing effect on the risk of CE. On the contrary, feeding UPCD a couple of times in a week during YO significantly (< 0.05) increased the risk of CE.

**Table 3:** Odds ratios (OR) of the associations between the puppyhood and adolescence diet style scores derived from principal component analysis and the incidence of chronic enteropathy (CE) in adult dogs based on multivariate logistic regression analyses (adjusted for dog gender).

	OR	95% CI		*p* value	n	
		Lower	Upper		Controls	Cases
**Puppyhood diet**						
RC1 NPMD + UPCD-	**0.777**	0.708	0.852	**0.0000000832**	3665	1016
RC1 NPMD-UPCD +	**1.287**	1.174	1.412	**0.0000000832**		
RC2 Cooked	1.056	0.977	1.141	ns (0.168494)		
RC3 Leftovers	**0.773**	0.714	0.837	**0.0000000002**		
**Adolescence diet**						
RC1 NPMD + UPCD-	**0.873**	0.789	0.966	**0.0084530000**	3227	699
RC1 NPMD-UPCD +	**1.146**	1.035	1.268	**0.0084530000**		
RC2 Cooked	1.057	0.966	1.155	ns (0.225928)		
RC3 Leftovers	**0.760**	0.692	0.835	**0.0000000098**		

The inverse association of NPMD (non-processed meat-based diet) and UPCD (ultra-processed carbohydrate-based diet) in the RC1 component means that owners gave both foods in reciprocal manner so that when the amount of NPMD given for the dog was higher, the amount of UPCD given was lower and vice versa. CI = confidence interval, Cooked = Home-cooked diet, Leftovers = Human meal leftovers and table scraps, ns = not significant.

Significant values are in bold.

### Single food items eaten during puppyhood and adolescence influence the future chronic enteropathy incidence

**Figure 3:**
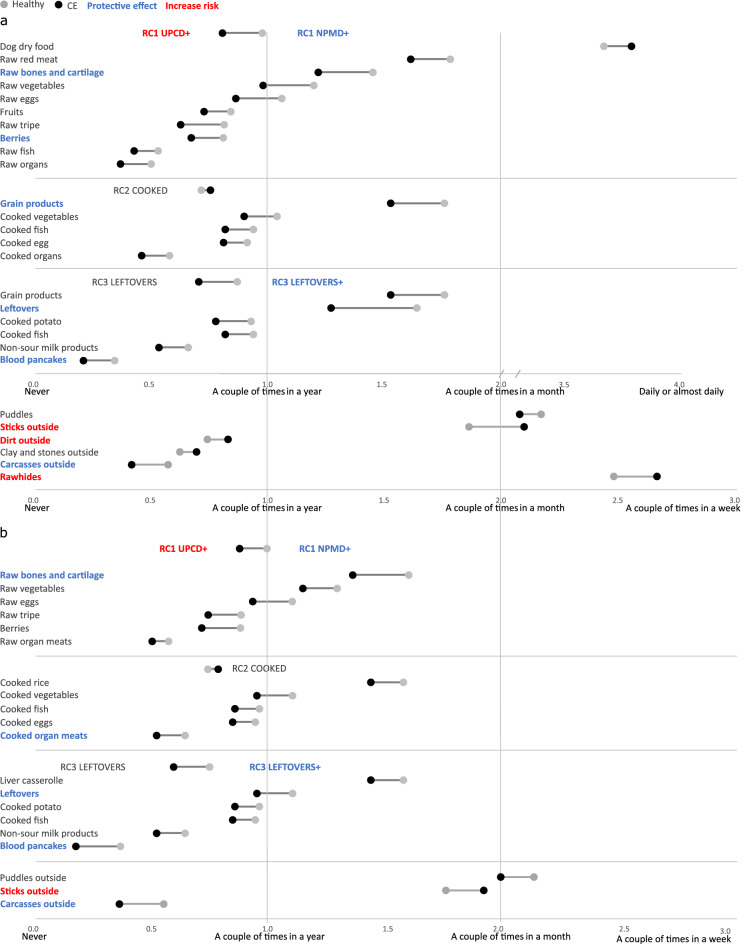
Dumbbell charts showing the diet style, food items within the diet style and other consumed items as mean feeding frequencies of control dogs (grey) and dogs with chronic enteropathy (CE; black) during a) puppyhood and b) adolescence. Only items that were significantly different (*p* < 0.05) between controls and cases in the preliminary screen are included. Diet and food items that significantly (*p* < 0.05) decreased the risk of CE later in life are highlighted as blue text. Diet and food items that significantly (*p* < 0.05) increased the risk of CE later in life are highlighted as red text. Text color and circles color legends are shown on the top of the figure. Detailed analysis results are shown in Table 3. Range values are shown in Supplementary information 3. NPMD = non-processed meat-based diet, UPCD = ultra-processed carbohydrate-based diet, COOKED = Home cooked diet, LEFTOVERS = Human meal leftovers and table scraps.

We analysed the single food items within the diet style PCA components (RC1, RC2, RC3) for association with CE later in life. Only items that were significantly different (*p* < 0.05) between controls and cases in the preliminary Mann–Whitney U-tests (Table 2 and Supplementary information 2) were included. In general control dogs ate non-processed food items more frequently than case dogs (Fig. 3). We found that raw bones and cartilage eaten during PU and YO were significantly associated with decreased CE incidence (Table 4). The effects were largest when raw bones and cartilage were given more than a couple of times per week during PU and a couple of times per week during YO (Table 4). In addition, berries eaten a couple of times per year during PU were significantly associated with decreased CE incidence (Table 4). Grain products eaten during PU and cooked organs eaten during YO within the component RC2 had a significant association with decreased future CE incidence (Table 4). Control dogs also ate more human meal leftovers and table scraps items than case dogs (Fig. 3). When the single food items of the RC3 component were analysed for association with future CE, leftovers and blood pancakes eaten during PU and YO were significantly associated with decreased incidence (Table 4). The effects were largest when leftovers were given more than a couple of times per week.

**Table 4:**
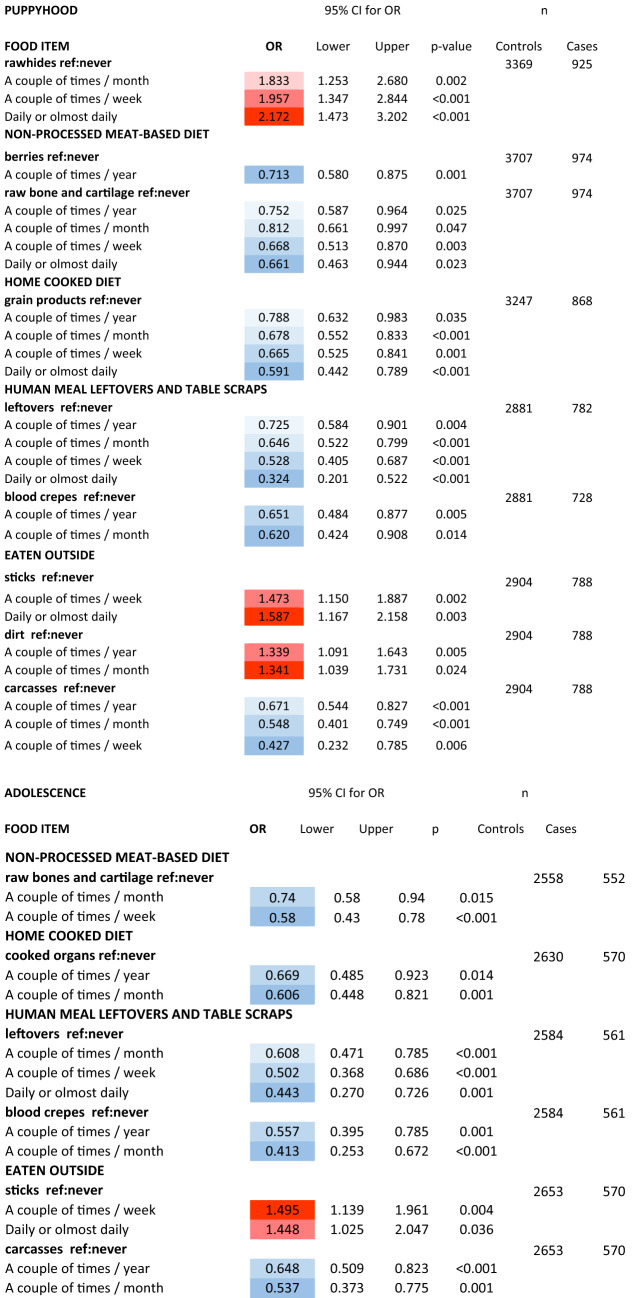
Odds ratios (OR) of the significant (*p* < 0.05) associations of food items within puppyhood or adolescent diet style and chronic enteropathy (CE) later in life, based on multivariate logistic regression analyses (adjusted for dog gender).

The OR cells are colored based on the values to ease reading and highlight the effect direction (red = increased risk, blue = protective effect) and increase in the effect size with increasing feeding frequency. CI = confidence interval, ref = reference.

Eating carcasses outside during PU and YO was significantly associated with decreased future CE incidence (Table 4, Fig. 3). In contrast, giving dogs rawhides (dried animal skin hide chews which have undergone various mechanical, chemical, and heat processing) during PU was associated with increased CE incidence in adulthood (Table 4, Fig. 3) and the risk increased with feeding frequency, being the highest, if rawhides were given more than a couple of times per week.

### Eating veterinary prescription dry dog food and indications of pica during puppyhood and adolescence are associated with future chronic enteropathy incidence

Part of case dogs ate gastrointestinal or allergy prescription dry dog food already during PU and YO and this was significantly associated with CE later in life (logistic regression PU *p* < 0.001, OR (CI) 1.578 (1.362–1.828) and YO *p* < 0.001, OR (CI) 1.373 (1.230–1.534)). This food variable was excluded from the further analyses because of likely reverse causality – food change after gastrointestinal symptoms appeared. Eating sticks outside a couple of times per week or more often compared to control dogs during PU and YO, and eating dirt outside more often compared to control dogs during PU, were significantly associated with increased incidence of CE (Table 4, Fig. 3).

## Discussion

Here, we studied the associations of companion dogs’ early life diet style with CE incidence in later life. Our results showed that feeding a non-processed or minimally processed meat-based diet to puppies and adolescent dogs at 2 to 18 months associated significantly with decreased CE incidence in adulthood. On the contrary, feeding an ultra-processed dry dog food (kibble) based diet associated significantly with increased CE incidence in adulthood. These findings support our previous observation that the consumption of a NPMD during PU may protect against CE later in life and that consumption of an UPCD increases CE incidence later in life^12^. These two diets differ essentially in their degree of processing^28^ and protein:fat:carbohydrate (PFC) macronutrient ratio. In NPMD the macronutrient ratio is typically 45:50:0–10 percent dry matter whereas in UPCD it is 16–38:6–18:40–60^29^.

The absence of a significant protective effect on CE later in life when feeding a home-cooked diet that is composed partly of similar, but cooked, food items as the non-processed meat-based diet, suggests that the lack of processing of the ingredients may be of importance. Dry dog food is ultra-processed by heat treatment, rendering, milling, and/or extrusion and contains food additives such as emulsifiers, colouring agents and palatability enhancers^28,30–33^. In contrast, meat-based non-processed foods for dogs are composed of fresh ingredients which may be chopped, mixed, and frozen and the only additives they might contain are minerals and vitamins if needed for balancing^34,35^. Thermal processing of foods containing carbohydrates and protein produces Maillard reaction products including advanced glycation end products (AGEs), which are immunomodulatory and may increase the prevalence of diet-related chronic inflammatory states in the gut^36,37^ although the current literature on the topic is partly controversial^38^. In addition, although the number of feed additives in ultra-processed carbohydrate-based dog food is vast, very little is known about their single or synergistic effects on gastrointestinal health^33,39,40^. Analogously, Western diet containing high amounts of ultra-processed foods and refined carbohydrates has been suggested as a potential explanation of increased prevalence of human IBD in the industrialized world^24^.

Domesticated dogs (*Canis familiaris*) are members of the order *Carnivora,* but can utilise non-animal foods such as plant material and carbohydrates in their diet^41,42^. However, only protein and fat are essential to the dog, they do not have dietary requirements for carbohydrates^43^. In addition, research indicates that dogs intuitively select a dietary macronutrient composition dominated by protein and fat^29,44^. Feeding dogs a NPMD is known to affect serum, urine and fecal metabolite concentrations when compared to feeding an UPCD (dry dog food) diet^45,46^ although the meaning of these changes still requires further research.

Finally, the intestinal bacterial flora plays a fundamental role in the pathogenesis of canine CE and human IBD^10,11,16,17^. The feeding of a NPMD has been shown to stimulate the growth of a balanced gastrointestinal microbiome in the dog, including an increase of the relative abundances of bacteria associated with protein and fat utilisation, in comparison to dogs fed an UPCD (dry dog food)^21,47^. Members of these genera also produce butyrate from protein and amino acid fermentation and thus contribute to the intestinal homeostasis. The diet is an essential part of the exposome^48^ and our observations are in accordance with the biodiversity hypothesis stating that the more microbial exposures in the early life, the more developed immune system in adulthood^12,49^. This may also explain why eating carcasses outside was negatively associated with CE later in life, contributing to the exposure of diverse microbiota. Fresh ingredients, including vegetables and roots as fibre and phytonutrient sources are part of the NPMD^34,35^. They contain a variety of microbes of which many may be beneficial for gut health^50^ as well as indigestible and soluble fibers which act as prebiotics and are fermented in the colon by bacteria to produce gut health promoting short-chain fatty acids^51–53^.

We further studied the associations of the consumed food items with CE incidence in later life. Out of the items belonging to the non-processed meat-based diet style, raw bones and cartilage and berries associated significantly with less CE in adulthood. The protective effect of raw bones and cartilage increased with increasing feeding frequency, being largest when fed daily or almost daily (PU) or a couple of times per week (YO). Eating raw bones and cartilage may have several benefits when fed to puppies and adolescent dogs. Chewing is a behavioural element of dog feeding^54–56^ and thus has been suggested to have a relaxing, stress-relieving effect and it also reduces chewing of unwanted objects when used as enrichment^57^. Chewing alone, however, does not explain the beneficial effect of bones and cartilage as consuming rawhides, that are chewing items as well, had a predisposing effect on CE incidence later in life as discussed later. Chewing raw bovine femur bones has also been shown effective in removing dental calculus in dogs^58,59^, but the same effect has been shown for “an extruded, chemically-coated rawhide preparation”^60^, and therefore cannot explain the beneficial effect of raw bones and cartilage either. While bone is composed primarily of inorganic calcium phosphate^61^, cartilage contains chondrocytes, collagen, glucosamine and proteoglycan aggregates that are made up of glycosaminoglycans, hyaluronic acid and chondroitin sulfate^62,63^. Glucosamine is a precursor for synthesis of the glycosaminoglycans and gastrointestinal glycoproteins (mucins) that are essential in mucosal defense^64^. Glucosamine and glycosaminoglycans modulate microbiota^65^. Glycosaminoglycans promote intestinal growth and wound repair^66^, have an effect on the inflammatory mediators and disease activity of IBD patients^67^, and may improve the intestinal membrane integrity of CE dogs^68^. Both collagen and collagen hydrolysate treatments have been shown to significantly reduce the mucosal damage score and facilitate faster regeneration of damaged GI mucosa^69^. Cartilage glycosaminoglycans have been shown to increase the uptake of iron from the diet, avoiding anemia – a symptom very commonly seen in CE^70,71^.

A whole prey-like diet including ground bone and cartilage as a source of animal fiber containing soluble and insoluble coarse may provide source substrates for gut health promoting short-chain fatty acid production by fermentation^72^. Mimicking an ancestral, whole-prey diet including bone and cartilage material, is also a possible explanation to the association of eating carcasses outside and less CE later in life.

Our results also indicated a protective role of eating berries against CE later in life. The berries eaten were mainly wild blueberries (*Vaccinium myrtillus*) which contain a variety of flavonoids, polyphenols, phenolic acids, pyruvic acid, chlorogenic acid, and other compounds which have anti-inflammatory, immunity enhancing and chronic disease preventing properties in humans as reviewed in Ma et al.^73^. Blueberries have been shown to have a significant antioxidant and anti-inflammatory activity when fed to dogs^74^. Blueberries were also shown to protect dogs against exercise-induced oxidative stress^74^. Moreover, in Finland berries are very often picked from the forest and many dogs even eat them directly from the plants in the forest. Therefore, these dogs also get exposed to the diverse forest microbiota which according to the biodiversity hypothesis promotes a healthy microbiome and a balanced immune system^4^. Eating berries during the berry picking season is a possible explanation why the answer “a couple of times in a year” was significantly different between the control and case dogs. Gray wolves (*Canis lupus*) consume berries seasonally when available or abundant^75^ and may even provision pups with them^76^.

Human meal leftovers and table scraps offered to the puppies and young dogs were found to be significantly associated with less CE later in life. The protective effect increased with feeding frequency, hence the more exposure the dogs had to leftovers, the more protection against CE development there was. Traditionally popular Finnish dishes and meals are composed of fish and meats, vegetables and roots, mushrooms, buttermilk and other fermented milk products, berries, and whole grain products, for example, black rye bread and oatmeal^77^. The raw meat scraps and the trimmings of the fatty parts might have a similar effect as the NPMD. Moreover, whole grain products as well as roots and vegetables contain indigestible and soluble fibres such as beta-glucans, which have gut health promoting effects^51,53,78–80^. This is a likely explanation for grain products showing a significant association with less CE in adulthood. Another explanation could be the popular Finnish dog food cooked at home; a long time soaked, slow-cooked porridge made of oats, millet, barley, buckwheat, and whole grain rice with vegetables and salt, often served with raw meat and eggs as the protein source (Finnish name Yrjölä porridge). Moreover, healthy humans might share mouth resident beneficial microbes^81^, like *Streptococcus salivarius*^82^, with their dogs, that are transferred to the puppies by contact with table utensils and hands. Furthermore, offering leftovers to dogs is indicative of a more intense human-canine bond, decreasing the stress a puppy experiences and affecting the immune system positively in both dogs and their owners^79,83–85^. Leftovers also decreased chronic gastrointestinal signs after acute gastric dilatation-volvulus surgery in Finnish dogs^86^.

Organ meats, that were found protective during YO within the home-cooked diet, contain vitamins and micronutrients like vitamins A and B, selenium and coenzyme Q10 which may have a positive impact on gut health^87–89^. It is not clear why blood pancakes were found protective; one possible explanation is blood as a protein and/or a source of iron.

Rawhides fed to puppies a couple of times in a month or more often was associated with an increased risk of CE later in life. The risk increased with increased feeding frequency being highest when rawhides were given more than one time per week during PU. This may be related to low digestibility of rawhides, measured as in vitro dry matter disappearance (DMD), which may pose a risk for gastrointestinal blockage and intolerance especially if the dog tends to swallow large size rawhide pieces which remain intact during gastric and intestinal phases^90^. However, as rawhides are made from leather industry by-products, it is also possible that the toxic chemicals used for leather processing cause the negative effects on gut health we saw in this study. Unfortunately, there are no scientific publications on analyses of rawhide composition available and clearly requires further research. In 2017, the Food and Drug Administration (FDA) announced a voluntary recall of rawhide chews for dogs due to the use of quaternary ammonia compounds during the rawhide processing^91^.

There is no conclusive data on the prevalence of CE in dogs, although the disease is frequently diagnosed at animal clinics worldwide (1–17.8%)^5^. The prevalence of owner-reported CE symptoms was 18–22% in the sample of Finnish FFQ responses. In this study, owners reported onset of CE symptoms at 1.4–1.5 years mean age. Part of the case dogs were also on gastrointestinal or allergy prescription dry dog food diets, commonly suggested by vets for dogs suffering from chronic GI symptoms^7^already at an early age as could be seen already from the PU and YO datasets. Moreover, eating sticks outside a couple of times per week or more often compared to control dogs during PU and YO, and eating dirt outside more than control dogs during PU, were associated with increased incidence of CE likely because of pica i.e. dogs seeking relief to discomfort or GI symptoms, or stress resulting from the pain^92,93^. By eating dirt it is also possible that dogs look for micronutrients^30,31^, prebiotics or lactobacilli, fulvic acid or some other nutrients that they are lacking. Eating sticks outside might be a reverse causality but may also result in mechanical damage in the gastrointestinal tract and possible perforations by the sticks’ sharp edges, which may cause inflammation. While symptoms have been noted in puppies and young dogs, CE is usually diagnosed later, in middle-aged dogs^94,95^. The difference between the onset of owner-reported symptoms in our data and the general reported age of diagnosis is likely explained by the progression of the disease from subtle to more severe clinical signs requiring veterinary care, and the time it takes to perform several tests for diagnosis (e.g. exclusion of other causes and histopathology of gut biopsies)^6^.

The current study has several strengths. The study data were obtained from a partially validated questionnaire which provides reasonable and trusted data. The study took reverse causality into account by excluding the dogs based on age criteria from analyses. A wide range of food items were covered in the questionnaire and the diet style was inferred from several food variables using multivariate methods. Single food items within the diet style and other edible items were also studied. Additionally, two age periods (puppyhood and adolescence) were included.

The present study has some limitations. The study design is an owner-reported longitudinal and cross-sectional study which only can suggest causal relationships, not an experimental study on cause-effect. An owner reported FFQ was used, which may have led to recall bias and misclassification of the food items. However, we have validated the owner's answers by resending the questionnaire for them to refill^26^. We can thus assume that recall bias was substantially reduced. The FFQ is based on frequencies and not quantity and therefore we could not estimate the amount of individual food items of interest. Also, due to the lack of details regarding the ingredients of the food variables, we were not able to examine the nutrient profiles of the diets. Many owners have left part of the detailed feeding frequency questions unanswered instead of giving the answer “never”. However, the sample size retained was of reasonable size. Unfortunately, no information on antibiotic treatments is available in the questionnaire used. Due to the complexity of this study (different diet styles, 47 individual food items and two life stages) we did not include environmental factors as they were discussed earlier in Hemida et al.^12^.

## Conclusions

World-wide, companion dogs are increasingly considered as family members, and consequently there is a growing focus on the health benefits of their diets. While studies on metabolome, microbiota, etc. have started to emerge, more scientific studies are needed to evaluate the overall benefits and risks of canine diet styles, especially regarding their impact on health and lifespan. Diet choices during puppyhood and adolescence are modifiable factors which, according to our results, might lessen or increase CE incidence later in the dog’s life. Our study provides proactive dog owners with information on healthy diets and of what food items to use and to avoid. The key findings from the present study confirm the tested hypothesis as we found a significant association between companion dog puppyhood and adolescence diet and the tendency to develop CE in adulthood. Feeding NPMD, even as an addition to UPCD, and giving the dog human meal leftovers and table scraps were found to be protective against CE later in life. Especially raw bones and cartilage, berries and leftovers were found to be beneficial. Therefore, we conclude that providing a variety of fresh, “real” foods for the dog especially during puppyhood, but also at young age, was identified as a significant potential protective factor of CE incidence later in life. On the contrary, feeding mainly or exclusively UPCD, namely dry dog food or “kibble”, during puppyhood and adolescence, or rawhides at least a couple of times in a month during puppyhood were found to be significant potential risk factors for CE later in life. A home-cooked diet was not significantly associated with CE incidence later in life in this study.

## Supplementary Information

Supplementary Information 1.

Supplementary Information 2.

Supplementary Information 3.
