# Supplementary material for: The effect of puppyhood and adolescent diet on the incidence of chronic enteropathy in dogs later in life
Source: Sci Rep. 2023 Feb 9;13:1830. doi: 10.1038/s41598-023-27866-z (PMC9911636; doi:10.1038/s41598-023-27866-z)
Supplement: Supplementary file 1 — Supplementary Information 1. [file 41598_2023_27866_MOESM1_ESM.docx]

| Supplementary Table S1. The food variables are same for both PU/YO. Grey text = more than 50% missing, was not used in the analyses. Bold text= included in the energy containing foods filter variable. | | |
| --- | --- | --- |
| **Variable name** | **Additional definition** | Type of original variables, number of answers |
|  |  |  |
| Egg shells |  | dropdown with frequency |
| **Cooked bone and cartilage** |  | frequency, dropdown with frequency |
| **Cooked eggs** |  | dropdown with frequency |
| **Cooked fish** |  | dropdown with frequency |
| **Cooked organ meats** |  | dropdown with frequency, 2 answers |
| **Cooked poultry** |  | dropdown with frequency |
| **Cooked red meat** |  | dropdown with frequency |
| **Processed meat** |  | dropdown with frequency |
| **Cooked tripe** |  | frequency |
| **Dry dog food** | Ultra-processed carbohydrate-based food, "kibble" | dropdown with frequency |
| **Prescription dry dog food** | Ultra-processed carbohydrate-based food, "kibble", for specific diseases. Sold mainly in veterinary practices | dropdown with frequency |
| Rawhides | Dried animal skin hide chews which have undergone various mechanical, chemical and heat processing | frequency |
| Treats | Processed dog treats including dental products, dog dry food as treats | frequency |
| Dried animal parts |  | frequency, dropdown with frequency |
| **Dried fish** |  | dropdown with frequency |
| **Wet dog food** |  | dropdown with frequency |
| Prescription wet dog food |  | dropdown with frequency |
| **Blood pancakes** |  | frequency |
| **Liver casserole** |  | frequncy, dropdown with frequency |
|  |  |  |
| Animal fats | Butter, tallow, lard, other animal fats excluding oils and dog dry food ingredients | dropdown with frequency |
| Fish oils |  | dropdown with frequency |
| Vegetable oils | Lindseed, hemp, rapeseed etc. oil supplemets | dropdown with frequency |
| Mixed oils | Mixes of vegetable and fish oils | dropdown with frequency |
|  |  |  |
| Fruits |  | dropdown with frequency, 2 answers |
| Cooked rice |  | frequncy, dropdown with frequency |
| Cooked vegetables |  | dropdown with frequency, 2 answers |
| Cooked potato |  | potatoes: frequency, dropdown with frequency |
| Grain products | Cooked porridge, grain, pasta | frequncy, dropdown with frequency |
| Seeds and nuts |  | dropdown with frequency |
| **Human meal leftovers** |  | frequency |
|  |  |  |
| Raw bone and cartilage |  | frequncy, dropdown with frequency |
| **Raw eggs** | Raw eggs except shells | dropdown with frequency |
| **Raw fish** |  | dropdown with frequency |
| **Raw organ meats** |  | dropdown with frequency, 2 answers |
| Raw poultry |  | dropdown with frequency |
| **Raw red meat** |  | dropdown with frequency |
| **Raw tripe** |  | frequency |
| Raw vegetables |  | dropdown with frequency, 2 answers |
| Raw berries |  | dropdown with frequency, 2 answers |
|  |  |  |
| Cheese |  | dropdown with frequency |
| **Non-sour milk products** | Eg. Milk, ice cream, cream. | dropdown with frequency, 2 answers |
| **Sour milk products** | Viili, youghurt, sour milk etc. | dropdown with frequency, 2 answers |
|  |  |  |
| Unspecified cooked meat | The meat source not specified | dropdown with frequency |
| Unspecified dog freshfood | The type of freshfood not specified | dropdown with frequency |
| Unspecified raw meat | The meat source not specified | dropdown with frequency |
| **Redmeat unknown** | The meat source and processing method not specified | dropdown with frequency |
|  |  |  |
| Carcasses outside |  | frequency |
| Clay and stones outside |  | frequency |
| Dirt outside |  | frequency |
| Puddles outside |  | frequency |
| Sticks outside |  | frequency |
| Grass outside |  | frequency |
| Feces outside |  | dropdown with frequency, two answers |
