# Supplementary material for: The effect of puppyhood and adolescent diet on the incidence of chronic enteropathy in dogs later in life
Source: Sci Rep. 2023 Feb 9;13:1830. doi: 10.1038/s41598-023-27866-z (PMC9911636; doi:10.1038/s41598-023-27866-z)
Supplement: Supplementary file 2 — Supplementary Information 2. [file 41598_2023_27866_MOESM2_ESM.docx]

Supplementary Table S2. Mann-Whitney U-test p-values and multiple comparison corrected q- and local fdr values for food items. Food items that were significantly (p<0.05) different between case and control groups are shown bolded black text and items p>0.05 gray normal text.

| Puppyhood food variables | Mann-Whitney U-test p-value | q-value | local fdr |
| --- | --- | --- | --- |
| **Human meal leftovers** | 0.000000 | 0.000000 | 0.000000 |
| **Prescription dry dog food** | 0.000000 | 0.000000 | 0.000000 |
| **Blood pancakes** | 0.000000 | 0.000000 | 0.000000 |
| **Carcasses outside** | 0.000000 | 0.000000 | 0.000002 |
| **Sticks outside** | 0.000001 | 0.000001 | 0.000003 |
| **Raw bone and cartilage** | 0.000002 | 0.000001 | 0.000006 |
| **Grain products** | 0.000003 | 0.000002 | 0.000009 |
| **Raw egg** | 0.000007 | 0.000003 | 0.000019 |
| **Cooked organ meats** | 0.000008 | 0.000003 | 0.000021 |
| **Raw vegetables** | 0.000021 | 0.000008 | 0.000053 |
| **Non-sour milk products** | 0.000024 | 0.000008 | 0.000059 |
| **Rawhides** | 0.000028 | 0.000009 | 0.000067 |
| **Raw berries** | 0.000084 | 0.000024 | 0.000180 |
| **Raw tripe** | 0.000091 | 0.000025 | 0.000193 |
| **Cooked potato** | 0.000202 | 0.000051 | 0.000397 |
| **Raw organ meats** | 0.000274 | 0.000065 | 0.000522 |
| **Cooked vegetables** | 0.001249 | 0.000279 | 0.002047 |
| **Dry dog food** | 0.001684 | 0.000356 | 0.002678 |
| **Cooked fish** | 0.001854 | 0.000371 | 0.002920 |
| **Redmeat unknown** | 0.002164 | 0.000412 | 0.003355 |
| **Raw fish** | 0.002687 | 0.000487 | 0.004076 |
| **Raw redmeat** | 0.004714 | 0.000815 | 0.006753 |
| **Cooked egg** | 0.005704 | 0.000943 | 0.008013 |
| **Animal fats** | 0.006966 | 0.001104 | 0.009586 |
| **Dried fish** | 0.010952 | 0.001666 | 0.014378 |
| **Fruits** | 0.013326 | 0.001947 | 0.017135 |
| **Dirt outside** | 0.013820 | 0.001947 | 0.017701 |
| **Clay and stones outside** | 0.036037 | 0.004896 | 0.041529 |
| **Wet dog food** | 0.043922 | 0.005761 | 0.049449 |
| **Puddles outside** | 0.045985 | 0.005831 | 0.051487 |
| Liver casserole | 0.052395 | 0.006349 | 0.057735 |
| Grass outside | 0.053406 | 0.006349 | 0.058711 |
| Sour milk products | 0.063017 | 0.007264 | 0.067840 |
| Fish oils | 0.093993 | 0.010516 | 0.095805 |
| Cooked rice | 0.158115 | 0.017185 | 0.148101 |
| Treats | 0.266662 | 0.028177 | 0.222899 |
| Dried animal parts | 0.294291 | 0.030256 | 0.239463 |
| Vegetable oils | 0.359837 | 0.036022 | 0.274908 |
| Cooked tripe | 0.434077 | 0.042339 | 0.308510 |
| Mixed oils | 0.513136 | 0.047669 | 0.336394 |
| Cooked poultry | 0.513787 | 0.047669 | 0.336588 |
| Cooked bone and cartilage | 0.640436 | 0.058005 | 0.362281 |
| Processed meat | 0.678497 | 0.060023 | 0.364733 |
| Feces outside | 0.851888 | 0.073457 | 0.364733 |
| Cooked redmeat | 0.868968 | 0.073457 | 0.364733 |
|  |  |  |  |
| Adolescent food variables | Mann-Whitney U-test p-value | q-value | local fdr |
| **Human meal leftovers** | 5.10703E-15 | 3.68E-14 | 1.91E-07 |
| **Prescription dry dog food** | 1.26321E-12 | 4.55E-12 | 1.91E-07 |
| **Blood pancakes** | 3.25144E-10 | 7.81E-10 | 1.91E-07 |
| **Carcasses outside** | 8.2976E-08 | 1.49E-07 | 1.35E-06 |
| **Cooked potato** | 3.56709E-07 | 5.14E-07 | 5.31E-06 |
| **Raw bone and cartilage** | 4.14162E-05 | 4.97E-05 | 0.000377 |
| **Raw berries** | 0.000144449 | 0.000149 | 0.00102 |
| **Raw egg** | 0.000776268 | 0.000669 | 0.003675 |
| **Cooked organ meats** | 0.00083544 | 0.000669 | 0.003883 |
| **Non-sour milk products** | 0.00156728 | 0.001129 | 0.006209 |
| **Sticks outside** | 0.003523728 | 0.002307 | 0.011321 |
| **Cooked vegetables** | 0.004298382 | 2.58E-03 | 1.31E-02 |
| **Raw tripe** | 0.008441699 | 0.004405 | 0.021558 |
| **Liver casserole** | 0.00856252 | 0.004405 | 0.021784 |
| **Raw vegetables** | 0.010385957 | 0.004987 | 0.025103 |
| **Cooked rice** | 0.01400691 | 0.006305 | 0.031259 |
| **Puddles outside** | 0.022409883 | 0.009495 | 0.04407 |
| **Cooked fish** | 0.029632503 | 0.011857 | 0.054014 |
| **Raw organ meats** | 0.040650085 | 0.01541 | 0.06795 |
| **Cooked egg** | 0.047128832 | 0.016973 | 0.075632 |
| Redmeat unknown | 0.050409006 | 0.016998 | 0.079404 |
| Feces outside | 0.051919428 | 1.70E-02 | 8.11E-02 |
| Raw redmeat | 0.054678371 | 0.017123 | 0.08421 |
| Processed meat | 0.122082258 | 0.035654 | 0.150044 |
| Fish oils | 0.125852658 | 0.035654 | 0.153345 |
| Animal fats | 0.12870353 | 0.035654 | 0.155822 |
| Rawhides | 0.141950785 | 0.037867 | 0.167121 |
| Grass outside | 0.185023763 | 0.046935 | 0.201838 |
| Raw fish | 0.191634652 | 0.046935 | 0.20693 |
| Dry dog food | 0.201112916 | 0.046935 | 0.214135 |
| Fruits | 0.213917728 | 0.046935 | 0.223694 |
| Treats | 0.214438784 | 0.046935 | 0.224079 |
| Sour milk products | 0.215040361 | 0.046935 | 0.224524 |
| Dried fish | 0.263097449 | 5.57E-02 | 2.59E-01 |
| Cooked poultry | 0.362051837 | 0.074507 | 0.322282 |
| Clay and stones outside | 0.380814682 | 0.076191 | 0.333346 |
| Wet dog food | 0.399406813 | 7.78E-02 | 3.44E-01 |
| Cooked bone and cartilage | 0.463691825 | 8.79E-02 | 0.378349 |
| Dirt outside | 0.478152959 | 0.088307 | 0.385513 |
| Vegetable oils | 0.591583407 | 0.106524 | 0.433247 |
| Cooked redmeat | 0.761324364 | 0.130969 | 0.463411 |
| Dried animal parts | 0.763704841 | 0.130969 | 0.463411 |
| Grain products | 0.817071358 | 0.136862 | 0.463411 |
| Mixed oils | 0.845014101 | 0.138326 | 0.463411 |
| Cooked tripe | 0.943399865 | 0.150999 | 0.463411 |
