# Supplementary material for: The effect of puppyhood and adolescent diet on the incidence of chronic enteropathy in dogs later in life
Source: Sci Rep. 2023 Feb 9;13:1830. doi: 10.1038/s41598-023-27866-z (PMC9911636; doi:10.1038/s41598-023-27866-z)
Supplement: Supplementary file 3 — Supplementary Information 3. [file 41598_2023_27866_MOESM3_ESM.docx]

Supplementary Table S3. Range values in Figure 3.

| \| PU \|  \| HEALTHY \| CE \|  \| YO \|  \| HEALTHY \| CE \| \| --- \| --- \| --- \| --- \| --- \| --- \| --- \| --- \| --- \| \|  \| RC1 NPMD-UPCD+ \| 0.98 \| 0.81 \|  \|  \| RC1 NPMD-UPCD+ \| 0.99 \| 0.87 \| \|  \| Dog dry food \| 3.66 \| 3.78 \|  \|  \|  \|  \|  \| \|  \| Raw red meat \| 1.78 \| 1.61 \|  \|  \| Raw bones and cartilage \| 1.59 \| 1.35 \| \|  \| Raw bones and cartilage \| 1.45 \| 1.22 \|  \|  \| Raw vegetables \| 1.28 \| 1.14 \| \|  \| Raw vegetables \| 1.20 \| 0.98 \|  \|  \| Raw eggs \| 1.10 \| 0.93 \| \|  \| Raw eggs \| 1.06 \| 0.86 \|  \|  \| Raw tripe \| 0.88 \| 0.74 \| \|  \| Fruits \| 0.84 \| 0.73 \|  \|  \| Berries \| 0.88 \| 0.71 \| \|  \| Raw tripe \| 0.81 \| 0.63 \|  \|  \| Raw organ meats \| 0.57 \| 0.50 \| \|  \| Berries \| 0.81 \| 0.67 \|  \|  \|  \|  \|  \| \|  \| Raw fish \| 0.53 \| 0.43 \|  \|  \|  \|  \|  \| \|  \| Raw organs \| 0.50 \| 0.37 \|  \|  \|  \|  \|  \| \|  \|  \|  \|  \|  \|  \|  \|  \|  \| \|  \| RC2 COOKED \| 0.71 \| 0.75 \|  \|  \| RC2 COOKED \| 0.74 \| 0.78 \| \|  \| Grain products \| 1.76 \| 1.53 \|  \|  \| Cooked rice \| 1.56 \| 1.43 \| \|  \| Cooked vegetables \| 1.04 \| 0.90 \|  \|  \| Cooked vegetables \| 1.10 \| 0.95 \| \|  \| Cooked fish \| 0.94 \| 0.82 \|  \|  \| Cooked fish \| 0.96 \| 0.85 \| \|  \| Cooked egg \| 0.91 \| 0.81 \|  \|  \| Cooked eggs \| 0.94 \| 0.84 \| \|  \| Cooked organs \| 0.58 \| 0.46 \|  \|  \| Cooked organ meats \| 0.64 \| 0.52 \| \|  \|  \|  \|  \|  \|  \|  \|  \|  \| \|  \| RC3 LEFTOVERS \| 0.87 \| 0.70 \|  \|  \| RC3 LEFTOVERS \| 0.75 \| 0.59 \| \|  \| Grain products \| 1.76 \| 1.53 \|  \|  \| Liver casserolle \| 1.56 \| 1.43 \| \|  \| Leftovers \| 1.64 \| 1.27 \|  \|  \| Leftovers \| 1.10 \| 0.95 \| \|  \| Cooked potato \| 0.93 \| 0.78 \|  \|  \| Cooked potato \| 0.96 \| 0.85 \| \|  \| Cooked fish \| 0.94 \| 0.82 \|  \|  \| Cooked fish \| 0.94 \| 0.84 \| \|  \| Non-sour milk products \| 0.66 \| 0.53 \|  \|  \| Non-sour milk products \| 0.64 \| 0.52 \| \|  \| Blood pancakes \| 0.34 \| 0.21 \|  \|  \| Blood pancakes \| 0.37 \| 0.18 \| \|  \|  \|  \|  \|  \|  \|  \|  \|  \| \|  \| OUTSIDE \|  \|  \|  \|  \| OUTSIDE \|  \|  \| \|  \| Puddles outside \| 2.16 \| 2.07 \|  \|  \| Puddles outside \| 2.12 \| 2.00 \| \|  \| Sticks outside \| 1.85 \| 2.09 \|  \|  \| Sticks outside \| 1.76 \| 1.92 \| \|  \| Dirt outside \| 0.73 \| 0.82 \|  \|  \| Carcasses outside \| 0.57 \| 0.38 \| \|  \| Clay and stones outside \| 0.62 \| 0.69 \|  \|  \|  \|  \|  \| \|  \| Carcasses outside \| 0.57 \| 0.41 \|  \|  \|  \|  \|  \| \|  \|  \|  \|  \|  \|  \|  \|  \|  \| \|  \| Rawhides \| 2.47 \| 2.66 \|  \|  \|  \|  \|  \| |  |  |  |  |  |  |  |  |
| --- | --- | --- | --- | --- | --- | --- | --- | --- | --- | --- | --- | --- | --- | --- | --- | --- | --- | --- | --- | --- | --- | --- | --- | --- | --- | --- | --- | --- | --- | --- | --- | --- | --- | --- | --- | --- | --- | --- | --- | --- | --- | --- | --- | --- | --- | --- | --- | --- | --- | --- | --- | --- | --- | --- | --- | --- | --- | --- | --- | --- | --- | --- | --- | --- | --- | --- | --- | --- | --- | --- | --- | --- | --- | --- | --- | --- | --- | --- | --- | --- | --- | --- | --- | --- | --- | --- | --- | --- | --- | --- | --- | --- | --- | --- | --- | --- | --- | --- | --- | --- | --- | --- | --- | --- | --- | --- | --- | --- | --- | --- | --- | --- | --- | --- | --- | --- | --- | --- | --- | --- | --- | --- | --- | --- | --- | --- | --- | --- | --- | --- | --- | --- | --- | --- | --- | --- | --- | --- | --- | --- | --- | --- | --- | --- | --- | --- | --- | --- | --- | --- | --- | --- | --- | --- | --- | --- | --- | --- | --- | --- | --- | --- | --- | --- | --- | --- | --- | --- | --- | --- | --- | --- | --- | --- | --- | --- | --- | --- | --- | --- | --- | --- | --- | --- | --- | --- | --- | --- | --- | --- | --- | --- | --- | --- | --- | --- | --- | --- | --- | --- | --- | --- | --- | --- | --- | --- | --- | --- | --- | --- | --- | --- | --- | --- | --- | --- | --- | --- | --- | --- | --- | --- | --- | --- | --- | --- | --- | --- | --- | --- | --- | --- | --- | --- | --- | --- | --- | --- | --- | --- | --- | --- | --- | --- | --- | --- | --- | --- | --- | --- | --- | --- | --- | --- | --- | --- | --- | --- | --- | --- | --- | --- | --- | --- | --- | --- | --- | --- | --- | --- | --- | --- | --- | --- | --- | --- | --- | --- | --- | --- | --- | --- | --- | --- | --- | --- | --- | --- | --- | --- | --- | --- | --- | --- | --- | --- | --- | --- | --- | --- | --- | --- | --- | --- | --- | --- | --- | --- | --- | --- | --- | --- | --- | --- | --- | --- | --- | --- | --- | --- | --- | --- | --- | --- | --- | --- | --- | --- | --- | --- | --- | --- |
|  |  |  |  |  |  |  |  |  |
|  |  |  |  |  |  |  |  |  |
|  |  |  |  |  |  |  |  |  |
|  |  |  |  |  |  |  |  |  |
|  |  |  |  |  |  |  |  |  |
|  |  |  |  |  |  |  |  |  |
|  |  |  |  |  |  |  |  |  |
|  |  |  |  |  |  |  |  |  |
|  |  |  |  |  |  |  |  |  |
|  |  |  |  |  |  |  |  |  |
|  |  |  |  |  |  |  |  |  |
|  |  |  |  |  |  |  |  |  |
|  |  |  |  |  |  |  |  |  |
|  |  |  |  |  |  |  |  |  |
|  |  |  |  |  |  |  |  |  |
|  |  |  |  |  |  |  |  |  |
|  |  |  |  |  |  |  |  |  |
|  |  |  |  |  |  |  |  |  |
|  |  |  |  |  |  |  |  |  |
|  |  |  |  |  |  |  |  |  |
|  |  |  |  |  |  |  |  |  |
|  |  |  |  |  |  |  |  |  |
|  |  |  |  |  |  |  |  |  |
|  |  |  |  |  |  |  |  |  |
|  |  |  |  |  |  |  |  |  |
|  |  |  |  |  |  |  |  |  |
|  |  |  |  |  |  |  |  |  |
|  |  |  |  |  |  |  |  |  |
|  |  |  |  |  |  |  |  |  |
|  |  |  |  |  |  |  |  |  |
|  |  |  |  |  |  |  |  |  |
|  |  |  |  |  |  |  |  |  |
|  |  |  |  |  |  |  |  |  |
|  |  |  |  |  |  |  |  |  |
|  |  |  |  |  |  |  |  |  |
